# Changes in the triglyceride glucose-body mass index estimate the risk of stroke in middle-aged and older Chinese adults: a nationwide prospective cohort study

**DOI:** 10.1186/s12933-023-01983-5

**Published:** 2023-09-16

**Authors:** Rong-Rui Huo, Lu Zhai, Qian Liao, Xue-Mei You

**Affiliations:** 1 Department of Experimental Research, Guangxi Medical University Cancer Hospital, He Di Rd. #71, Nanning, 530021 China; 2 Department of Smart Health Elderly Care Services and Management, School of Nursing, Guangxi Health Science College, Nanning, China; 3 Department of Epidemiology and Health Statistics, School of Public Health, Guangxi Medical University, Nanning, 530021 Guangxi China; 4 Key Laboratory of Early Prevention and Treatment for Regional High Frequency Tumour (Guangxi Medical University), Ministry of Education, Nanning, 530021 China; 5 Guangxi Key Laboratory of Early Prevention and Treatment for Regional High Frequency Tumour, Nanning, 530021 China

**Keywords:** Stroke, Long-term changes, Triglyceride glucose-body mass index, K-means clustering, CHARLS

## Abstract

**Background:**

Stroke was reported to be highly correlated with the triglyceride glucose-body mass index (TyG-BMI). Nevertheless, literature exploring the association between changes in the TyG-BMI and stroke incidence is scant, with most studies focusing on individual values of the TyG-BMI. We aimed to investigate whether changes in the TyG-BMI were associated with stroke incidence.

**Methods:**

Data were obtained from the China Health and Retirement Longitudinal Study (CHARLS), which is an ongoing nationally representative prospective cohort study. The exposures were changes in the TyG-BMI and cumulative TyG-BMI from 2012 to 2015. Changes in the TyG-BMI were classified using K-means clustering analysis, and the cumulative TyG-BMI was calculated as follows: (TyG-BMI_2012_ + TyG-BMI_2015_)/2 × time (2015–2012). Logistic regressions were used to determine the association between different TyG-BMI change classes and stroke incidence. Meanwhile, restricted cubic spline regression was applied to examine the potential nonlinear association of the cumulative TyG-BMI and stroke incidence. Weighted quantile sum regression was used to provide a comprehensive explanation of the TyG-BMI by calculating the weights of FBG, triglyceride-glucose (TG), and BMI.

**Results:**

Of the 4583 participants (mean [SD] age at baseline, 58.68 [9.51] years), 2026 (44.9%) were men. During the 3 years of follow-up, 277 (6.0%) incident stroke cases were identified. After adjusting for potential confounders, compared to the participants with a consistently low TyG-BMI, the OR for a moderate TyG-BMI with a slow rising trend was 1.01 (95% CI 0.65–1.57), the OR for a high TyG-BMI with a slow rising trend was 1.62 (95% CI 1.11–2.32), and the OR for the highest TyG-BMI with a slow declining trend was 1.71 (95% CI 1.01–2.89). The association between the cumulative TyG-BMI and stroke risk was nonlinear (P_association_ = 0.017; P_nonlinearity_ = 0.012). TG emerged as the primary contributor when the weights were assigned to the constituent elements of the TyG-BMI (weight_2012_ = 0.466; weight_2015_ = 0.530).

**Conclusions:**

Substantial changes in the TyG-BMI are independently associated with the risk of stroke in middle-aged and older adults. Monitoring long-term changes in the TyG-BMI may assist with the early identification of individuals at high risk of stroke.

**Supplementary Information:**

The online version contains supplementary material available at 10.1186/s12933-023-01983-5.

## Introduction

Stroke, the foremost cause of mortality and disability in numerous nations, poses a progressively escalating burden on the global healthcare system [1]. The Global Burden of Disease 2013 Study reported a substantial number of stroke survivors, with approximately 25.7 million individuals alive, along with 6.5 million stroke-related fatalities and 113 million stroke-related disability-adjusted life-years observed worldwide [2]. While the age-standardized mortality rate of stroke has exhibited a decline on a global scale, the incidence and prevalence of stroke in China continue to exhibit an alarming increase [2–4]. Consequently, there exists an immediate imperative to develop cost-effective and reproducible indicators that can enhance the stratification of stroke risk.

Insulin resistance (IR), recognized as a novel risk factor for stroke, is considered an early indication of type-2 diabetes, extending beyond patients with diabetes to include nondiabetic individuals as well [5]. Various methods exist to evaluate IR, with the hyperinsulinaemic glucose clamp (HEC) being the established reference standard [6]. However, HEC necessitates intravenous administration of glucose and insulin, accompanied by multiple blood samples [7]. This intricate and costly procedure is not widely employed in clinical practice. In contrast, the Homeostasis Model Assessment of IR (HOMA-IR) has gained widespread usage and demonstrated effectiveness in predicting cardiovascular and cerebrovascular diseases [7–10]. Nonetheless, HOMA-IR requires measurement of fasting insulin levels, which possesses limited clinical practicality. As a feasible alternative for assessing IR, the triglyceride-glucose (TyG) index can be effortlessly derived from routine clinical laboratory tests and is reported to be associated with stroke occurrence and recurrence. Furthermore, recent investigations have revealed that the TyG index outperforms HOMA-IR in predicting stroke [11, 12].

Recently, a study introduced a novel index called the triglyceride glucose-body mass index (TyG-BMI), which is computed as ln [TG (mg/dl) × FBG (mg/dl)/2] × BMI (kg/m^2^), with the objective of using it as a potential and straightforwards marker for IR [13]. TyG-BMI incorporates BMI into its calculation. This modification may allow TyG-BMI to better account for the influence of obesity on IR, and the combination of obesity and TyG can potentially identify IR more strongly than other surrogate markers, since obesity is a well-established risk factor for IR [14]. Several studies have also shown that TyG-BMI has a better predictive performance than TyG in metabolic diseases or cardiovascular disease [15–18]. However, only a cross-sectional study conducted on two population-based samples in China demonstrated a positive association between a high TyG-BMI and ischaemic stroke [19]. However, the exploration of the association between changes in TyG-BMI and stroke incidence has been infrequently reported in the literature, with most studies focusing on individual TyG-BMI values. Despite the valuable insights provided by earlier studies, there remains a notable gap in our understanding of how changes in TyG-BMI may relate to stroke risk, especially in a nationwide context. In addition, the relative contributions of TG, FBG, and BMI to stroke incidence is not clear. Our study aims to fill this gap by exploring this relationship in greater depth, utilizing data sourced from the "China Health and Retirement Longitudinal Study (CHARLS)", an ongoing nationwide cohort study designed to represent the population.

## Methods

### Study population

This study utilizes a secondary analysis of data from the CHARLS, an ongoing nationwide cohort study designed to represent the population [20]. The study design has been previously described [20]. In summary, a total of 17,708 participants residing in 10,257 households were selected using a multistage stratified probability-proportional-to-size sampling technique. These participants were recruited from 150 counties or districts and 450 villages within 28 provinces in China, spanning the period from June 2011 to March 2012. A standardized questionnaire was administered to collect information on sociodemographic and lifestyle factors, as well as health-related data. The baseline survey (Wave 1) achieved a response rate of 80.5%, and subsequent follow-up assessments were conducted every 2 years, with Wave 2 in 2013, Wave 3 in 2015 and Wave 4 in 2018. Blood samples were also collected at baseline and Wave 3. For this analysis, participants had to be aged 45 years and older, and complete data on fasting blood glucose (FBG), triglycerides (TG) and body mass index (BMI) were needed. People were excluded if they had a stroke before 2015.

The CHARLS study was approved by the institutional review board of Peking University. Written informed consent was obtained from all participants. This study was conducted following the Strengthening the Reporting of Observational Studies in Epidemiology (STROBE) reporting guidelines [21].

### Assessment of the change in TyG-BMI

The exposure of this study was the change in TyG-BMI values between 2012 and 2015. The TyG-BMI was calculated by the formula ln[TG (mg/dl) × FBG (mg/dl)/2] × BMI (kg/m^2^) [13]. We calculated the cumulative TyG-BMI with reference to the cumulative TyG change formula [22]: (TyG-BMI_2012_ + TyG-BMI_2015_)/2 × time (2015 − 2012). Height and weight were measured by a trained nurse. BMI was calculated as weight in kilograms divided by height in metres squared.

### Ascertainment of incident stroke events

Stroke was the main outcome of this study. In accordance with previous studies [23, 24], stroke events were assessed by individuals who self-reported “yes” to the question of “Have you been diagnosed with stroke by a doctor?” or selected specific answers to questions regarding the treatment of stroke “by Chinese Traditional Medicine/Western Modern Medicine/Physical Therapy/Acupuncture and Moxibustion/Occupational Therapy/None of the Above” were regarded as people with stroke. The date of stroke diagnosis was recorded as being between the date of the last interview and that of the interview reporting an incident stroke [23, 24].

### Covariates

At baseline (Wave 1), trained interviewers collected information on sociodemographic status and health-related factors using a structured questionnaire, including age, sex, living residence, marital status, and educational level. Educational level was classified as no formal education, primary school, middle or high school, and college or above. Health-related factors included self-reported smoking and drinking status (never, former, or current), self-reported physician-diagnosed medical conditions (diabetes, hypertension, heart problems, kidney disease, and dyslipidaemia), and use of medications for diabetes, hypertension, and dyslipidaemia. Laboratory examination contained total cholesterol (TC), high-density lipoprotein cholesterol (HDL-C), low-density lipoprotein cholesterol (LDL-C), estimated glomerular filtration ratio (eGFR), and glycosylated haemoglobin, type A1c (HbA1c) [25]. Marital status was classified into 2 groups: married and other marital status (never married, separated, divorced, and widowed). Diabetes was defined as fasting plasma glucose ≥ 126 mg/dl (to convert to millimoles per litre, multiplied by 0.0555), current use of antidiabetic medication, or self-reported history of diabetes. Hypertension was defined as systolic blood pressure ≥ 140 mmHg, diastolic blood pressure ≥ 90 mmHg, current use of antihypertensive medication, or self-reported history of hypertension. Dyslipidaemia was defined as total cholesterol ≥ 240 mg/dl (to convert to millimoles per litre, multiplied by 0.0259), triglycerides ≥ 150 mg/dl, low-density lipoprotein cholesterol ≥ 160 mg/dl, high-density lipoprotein cholesterol < 40 mg/dl, current use of lipid-lowering medication, or self-reported history of dyslipidaemia.

### Statistical analyses

Data were analysed using various statistical methods. For normally distributed continuous variables, means and standard deviations (SDs) were reported. Categorical variables were described in terms of frequency and percentage. The χ^2^ test and analysis of variance were utilized to compare differences in the baseline characteristics between the different groups, as appropriate. Approximately 4.3% (197/4583) of the total data items were found to be missing. These missing values were assumed to be missing at random and were addressed through the multiple imputation of chained equations method using the baseline characteristics. To account for the missing data, five imputed datasets were created. The results were then pooled using R statistical software along with the mice package.

We utilized an unsupervised machine learning technique called K-means with Euclidean distance to group patients based on their TyG-BMI measurements in 2012 and 2015. We opted for the k-means algorithm due to its computational efficiency and ability to generate easy-to-understand visualizations of the data points [26]. The K-means algorithm, a centroid-based clustering approach, divides a dataset into K clusters by minimizing the sum of squared distances within each cluster [26, 27]. To execute the process, we followed a three-step procedure: first, we specified the desired number of clusters; then, we randomly selected k patients as the initial cluster centres; next, we assigned each patient to the nearest centroid and sequentially updated the cluster centroids [27]. This iterative process continued until the total within-cluster sum of squares was minimized, and each patient was assigned to a specific cluster based on their distance from the centres, as determined by the Euclidean distance [26]. To determine the appropriate number of clusters, we visually analysed the reduction in the sum of squared distances resulting from varying the number of clusters. We presented a visual representation of the resulting clustering. Importantly, throughout the entire clustering process, the algorithm remained unaware of the outcome variables, ensuring unbiased analysis.

**Fig. 1:**
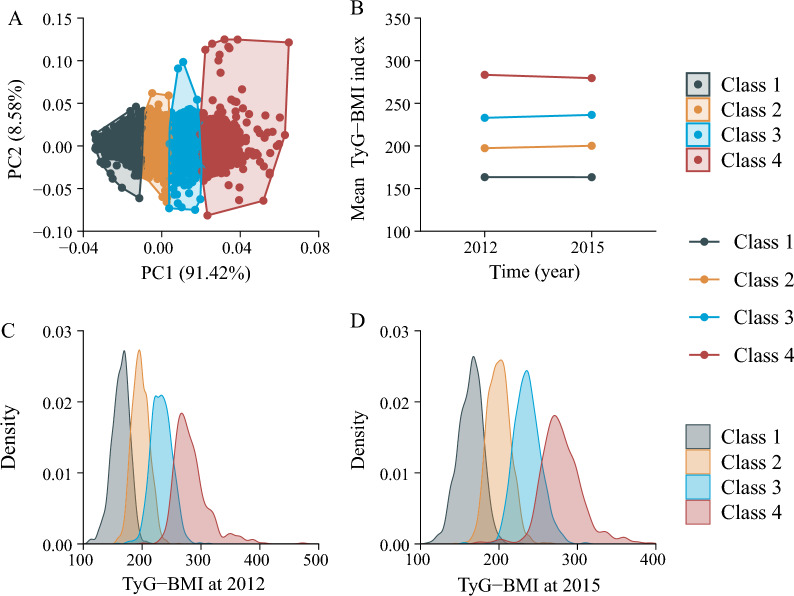
Clustering of the change in the TyG-BMI from 2012 to 2015. **A** Four clusters were found using the K-means method with Euclidean distance: the x- and y-axes are principal components of the change in the TyG-BMI; **B** data visualization for the classes of the change in the TyG-BMI: in Class 1 (n = 1273), the TyG-BMI ranged from 163.42 ± 15.13 in 2012 to 163.36 ± 15.45 in 2015 (P = 0.914); in Class 2 (n = 1664), the TyG-BMI ranged from 197.39 ± 14.40 in 2012 to 200.17 ± 14.45 in 2015 (P < 0.001); in Class 3 (n = 1171), the TyG-BMI ranged from 233.00 ± 17.59 in 2012 to 236.43 ± 16.93 in 2015 (P < 0.001); and in Class 4 (n = 475), the TyG-BMI ranged from 283.42 ± 29.91 in 2012 to 279.56 ± 27.16 in 2015 (P = 0.037); **C**, **D** Distribution for the TyG-BMI at 2012 or 2015: the normal distribution of the TyG-BMI within each class is observable, and there exists a statistically significant disparity in the mean TyG-BMI values among these classes. BMI: body mass index; PC: principal component; TyG: triglyceride-glucose

During a median follow-up of 36.7 months (range: 29.6–41.8 months) from baseline to Wave 3, the data set of transition of the TyG-BMI was analysed and classified into 4 classes using K-means clustering (Fig. 1A), and the paired t test was employed to assess the changes within each class: for Class 1 (n = 1273), the TyG-BMI ranged from 163.42 ± 15.13 in 2012 to 163.36 ± 15.45 in 2015 (P = 0.914), and the mean (SD) cumulative TyG-BMI was 490.18 ± 38.64, representing a consistently low TyG-BMI; for Class 2 (n = 1664), the TyG-BMI ranged from 197.39 ± 14.40 in 2012 to 200.17 ± 14.45 in 2015 (P < 0.001), and the mean (SD) cumulative TyG-BMI was 596.34 ± 30.55, representing a moderate TyG-BMI with a slow rising trend; for Class 3 (n = 1171), the TyG-BMI ranged from 233.00 ± 17.59 in 2012 to 236.43 ± 16.93 in 2015 (P < 0.001), and the mean (SD) cumulative TyG-BMI was 704.14 ± 34.37, representing a high TyG-BMI with a slow rising trend; for Class 4 (n = 475), the TyG-BMI ranged from 283.42 ± 29.91 in 2012 to 279.56 ± 27.16 in 2015 (P = 0.037), and the mean (SD) cumulative TyG-BMI was 844.48 ± 62.90, representing the highest TyG-BMI with a slow declining trend (Fig. 1B). The distribution of TyG-BMI according to the change in TyG-BMI classes is shown in Fig. 1C, D. The normal distribution of TyG-BMI within each class is observable, and there exists a statistically significant disparity in the mean TyG-BMI values among these classes.

Upon establishing classes of the change in TyG-BMI, we examined the association between different classes with changes in TyG-BMI and stroke events, and binary logistic models were used to calculate odds ratios (ORs) with 95% CIs. Four models were estimated: Model 1 adjusted for age and sex; Model 2 adjusted for age, sex, marital status, residence, educational level, smoking status, and drinking status; Model 3 adjusted for the variables in Model 2 and history of hypertension, diabetes, heart disease, dyslipidaemia, kidney disease, medication use for hypertension, medication use for diabetes, medication use for dyslipidaemia, systolic blood pressure, and diastolic blood pressure; and Model 4 adjusted for the variables in Model 3 and total cholesterol, HDL-C, LDL-C, HbA1c, and the eGFR.

To examine the association between the cumulative TyG-BMI and stroke events, the cumulative TyG-BMI was split into quartiles and then included in binary logistic models with the first quartile as the reference group. We searched for a linear trend by modelling the median value of each quantile to test ordered relations across quantiles of cumulative TyG-BMI. In addition, we explored the potential nonlinear association using a restricted cubic spline (RCS) regression model, and the model was conducted with 4 knots at the 5th, 35th, 65th, and 95th percentiles of cumulative TyG-BMI (reference is the 5th percentile). We further applied a two-piecewise linear regression model to examine the threshold effect of the cumulative TyG-BMI on stroke using a smoothing function [28, 29]. Subgroup analyses were conducted to examine whether the potential association of the change in TyG-BMI and cumulative TyG-BMI with stroke was moderated by the following demographic and clinical characteristics: age, sex, marital status, residence, educational level, smoking status, drinking status, history of hypertension, diabetes, heart disease, dyslipidaemia, and kidney disease. P values for interaction were evaluated using interaction terms and likelihood ratio tests.

To evaluate the performance of the TyG-BMI in predicting stroke, a receiver-operating characteristic (ROC) curve analysis was conducted, the area under the ROC curves (AUCs) were calculated, and the curves were generated with a smooth kernel density. To evaluate the extent to which TyG-BMI improved the prediction performance over TyG, Delong’s test was performed. TyG-BMI was derived using a mathematical formula incorporating TG, FBG, and BMI variables. To provide a comprehensive explanation of the formula, we employed the weighted quantile sum (WQS) regression model, employing bootstrap resampling methods for 1000 iterations. The WQS model facilitated the determination of weights assigned to FBG, TG, and BMI, quantifying their respective contributions to the overall effect. These weights were constrained within the range of 0 to 1, with a cumulative sum of 1 [30]. Higher weights indicated greater significance of the corresponding indicator in stroke prediction.

Five sensitivity analyses were conducted as follows: (1) repeating primary analyses using the complete data set (4373 participants) without multiple imputations; (2) repeating primary analyses excluding participants who had heart disease (538 participants) to account for loss to follow-up due to cardiovascular disease; (3) using the competing risk model to account for competing risks due to mortality (188 participant deaths); (4) using the Cox proportional hazards models to account for censored data; and (5) treating cumulative TyG-BMI as a continuous variable to examine the linear relationship between cumulative TyG-BMI and the risk of stroke without imposing predefined categories. Considering that the alteration in effect size per unit of TyG-BMI is small, we undertook the normalization of TyG-BMI to assess the effect in terms of a per-SD change in TyG-BMI. A two-sided P < 0.05 was considered to indicate statistical significance. All analyses were performed using R statistical software version 4.2.2 (R Foundation).

## Results

### Baseline characteristics of study participants

**Fig. 2:**
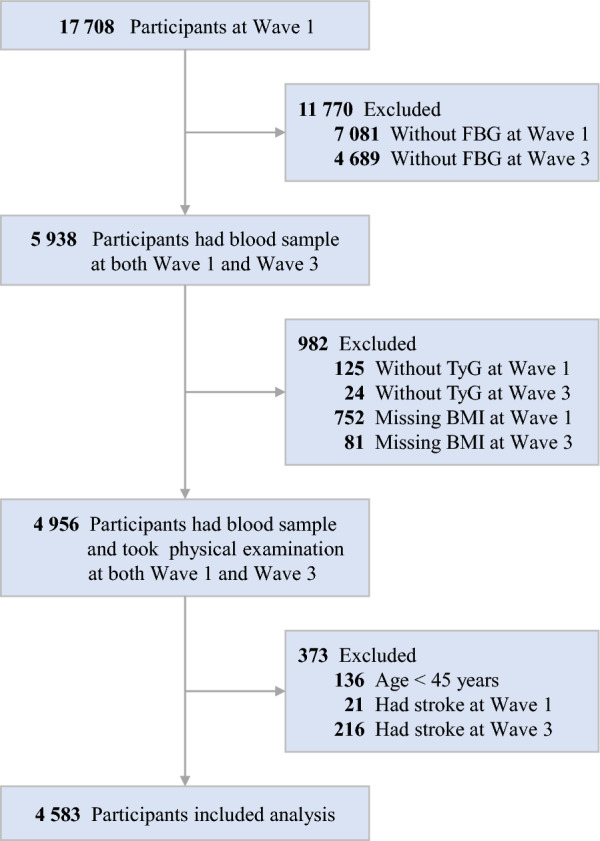
Flowchart of the study population. BMI: body mass index; FBG: fasting blood glucose; TyG: triglyceride-glucose

Of the 17,708 CHARLS participants at study baseline, we excluded 11,770 individuals who lacked FBG data at Waves 1 and 3. Additionally, 982 participants were excluded due to incomplete information on the TyG index and BMI at Waves 1 and 3. Furthermore, we excluded 136 participants younger than 45 years and 237 individuals who reported a history of stroke at Waves 1 and 3. Consequently, a total of 4583 participants met the inclusion criteria and were included in the subsequent analysis (Fig. 2). A comparison of baseline characteristics between participants included and those who were not included in the analysis is shown in Additional file 1: Table S1.

A total of 4583 adults were included in the analyses. The mean age at baseline was 58.68 ± 9.51 years; 2056 (44.9%) of the participants were men, and 2527 (55.1%) were women. Table 1 shows the characteristics of the participants. The mean TyG-BMI was 205.97 ± 40.60 in 2012 and 207.44 ± 40.06 in 2015, and the mean cumulative TyG-BMI was 620.12 ± 115.68.

**Table 1:** Baseline characteristics of 4583 participants according to the change in the TyG-BMI

Characteristic	Overall (n = 4583)	Change in the TyG-BMI				P value^a^
		Class 1 (n = 1273)	Class 2 (n = 1664)	Class 3 (n = 1171)	Class 4 (n = 475)	
Age, mean ± SD, years	58.68 ± 8.62	60.97 ± 8.94	58.50 ± 8.47	57.30 ± 8.26	56.55 ± 7.72	< 0.001
Sex						< 0.001
Male	2056 (44.9%)	743 (58.4%)	733 (44.1%)	424 (36.2%)	156 (32.8%)	
Female	2527 (55.1%)	530 (41.6%)	931 (55.9%)	747 (63.8%)	319 (67.2%)	
Marital status						< 0.001
Married	3905 (85.2%)	1052 (82.6%)	1407 (84.6%)	1012 (86.4%)	434 (91.4%)	
Other	678 (14.8%)	221 (17.4%)	257 (15.4%)	159 (13.6%)	41 (8.6%)	
Residence						< 0.001
Urban	1528 (33.3%)	301 (23.6%)	537 (32.3%)	481 (41.1%)	209 (44.0%)	
Rural	3055 (66.7%)	972 (76.4%)	1127 (67.7%)	690 (58.9%)	266 (56.0%)	
Educational level						< 0.001
No formal education	1344 (29.3%)	399 (31.3%)	492 (29.6%)	310 (26.5%)	143 (30.1%)	
Primary school	1910 (41.7%)	570 (44.8%)	680 (40.9%)	481 (41.1%)	179 (37.7%)	
Middle or high school	1207 (26.3%)	284 (22.3%)	440 (26.4%)	344 (29.4%)	139 (29.3%)	
College or above	122 (2.7%)	20 (1.6%)	52 (3.1%)	36 (3.1%)	14 (2.9%)	
Smoking status^b^						< 0.001
Never	2858 (62.4%)	627 (49.3%)	1067 (64.1%)	829 (70.8%)	335 (70.5%)	
Former	374 (8.2%)	97 (7.6%)	121 (7.3%)	112 (9.6%)	44 (9.3%)	
Current	1341 (29.3%)	546 (42.9%)	471 (28.3%)	228 (19.5%)	96 (20.2%)	
Drinking status^b^						< 0.001
Never	2700 (58.9%)	651 (51.1%)	993 (59.7%)	739 (63.1%)	317 (66.7%)	
Former	367 (8.0%)	122 (9.6%)	112 (6.7%)	104 (8.9%)	29 (6.1%)	
Current	1513 (33.0%)	499 (39.2%)	558 (33.5%)	327 (27.9%)	129 (27.2%)	
History of comorbidities						
Hypertension^b^	1216 (26.5%)	173 (13.6%)	371 (22.3%)	426 (36.4%)	246 (51.8%)	< 0.001
Diabetes^b^	281 (6.1%)	26 (2.0%)	74 (4.4%)	103 (8.8%)	78 (16.4%)	< 0.001
Heart disease^b^	537 (11.7%)	113 (8.9%)	164 (9.9%)	160 (13.7%)	100 (21.1%)	< 0.001
Dyslipidaemia^b^	491 (10.7%)	40 (3.1%)	142 (8.5%)	168 (14.3%)	141 (29.7%)	< 0.001
Kidney disease^b^	256 (5.6%)	70 (5.5%)	84 (5.0%)	75 (6.4%)	27 (5.7%)	0.493
History of medication use						
Hypertension medications^b^	885 (19.3%)	114 (9.0%)	249 (15.0%)	318 (27.2%)	204 (42.9%)	< 0.001
Diabetes medications^b^	171 (3.7%)	18 (1.4%)	38 (2.3%)	61 (5.2%)	54 (11.4%)	< 0.001
Dyslipidaemia medications^b^	257 (5.6%)	18 (1.4%)	65 (3.9%)	92 (7.9%)	82 (17.3%)	< 0.001
Blood pressure, mean ± SD, mmHg						
Systolic^b^	128.44 ± 20.63	123.79 ± 19.81	127.10 ± 19.89	132.10 ± 20.81	136.61 ± 20.99	< 0.001
Diastolic^b^	75.00 ± 11.98	71.39 ± 11.16	74.17 ± 11.53	77.84 ± 11.97	80.64 ± 12.08	< 0.001
TC, mean ± SD, mg/dl	194.74 ± 38.93	185.54 ± 36.22	193.49 ± 37.02	200.13 ± 37.91	210.52 ± 47.15	< 0.001
HDL-C, mean ± SD, mg/dl	51.25 ± 15.29	59.54 ± 16.26	52.23 ± 13.67	45.37 ± 12.09	40.09 ± 11.60	< 0.001
LDL-C, mean ± SD, mg/dl^b^	117.61 ± 34.97	110.75 ± 31.35	119.58 ± 33.44	122.30 ± 36.10	117.55 ± 43.13	< 0.001
HbA1c, mean ± SD	5.29 ± 0.81	5.14 ± 0.58	5.20 ± 0.65	5.40 ± 0.94	5.73 ± 1.20	< 0.001
eGFR, mean ± SD, ml/min/1.73 m^2b^	74.51 ± 33.82	73.36 ± 35.30	74.55 ± 32.57	74.89 ± 31.14	76.53 ± 39.88	0.433
TG_2012_, median (IQR)	104.43 (74.34, 152.22)	74.34 (58.41, 100.00)	100.00 (74.34, 137.18)	135.40 (100.45, 188.51)	193.82 (131.87, 286.30)	< 0.001
TG_2015_, median (IQR)	113.27 (81.42, 164.60)	80.53 (65.49, 105.31)	107.96 (83.19, 147.79)	150.44 (111.50, 207.08)	208.85 (142.92, 292.92)	< 0.001
FBG_2012_, mean ± SD	109.61 ± 34.26	102.11 ± 22.68	105.59 ± 24.95	114.56 ± 40.19	131.58 ± 55.19	< 0.001
FBG_2015_, mean ± SD	101.22 ± 31.66	92.45 ± 18.22	97.44 ± 23.01	107.74 ± 37.91	121.90 ± 51.01	< 0.001
BMI_2012_, mean ± SD	23.65 ± 3.77	19.79 ± 1.73	23.04 ± 1.62	26.08 ± 2.01	30.14 ± 3.42	< 0.001
BMI_2015_, mean ± SD	23.81 ± 3.71	19.83 ± 1.76	23.34 ± 1.66	26.37 ± 2.02	29.85 ± 2.96	< 0.001
TyG-BMI_2012_, mean ± SD^c^	205.97 ± 40.60	163.42 ± 15.13	197.39 ± 14.40	233.00 ± 17.59	283.42 ± 29.91	< 0.001
TyG-BMI_2015_, mean ± SD^c^	207.44 ± 40.06	163.36 ± 15.45	200.17 ± 14.45	236.43 ± 16.93	279.56 ± 27.16	< 0.001
Cumulative TyG-BMI index, mean ± SD^d^	620.12 ± 115.68	490.18 ± 38.64	596.34 ± 30.55	704.14 ± 34.37	844.48 ± 62.90	< 0.001

BMI: body mass index; eGFR: estimated glomerular filtration ratio; FBG: fast blood glucose; HbA1c: glycated haemoglobin; HDL-C: high-density lipoprotein cholesterol; IQR: interquartile range; LDL-C: low-density lipoprotein cholesterol; SD: standard deviation; TC: total cholesterol; TG: triglyceride; TyG: triglyceride-glucose

^a^P value was based on χ^2^ or analysis of variance test where appropriate

^b^Missing data: 10 for smoking status, 3 for drinking status, 23 for hypertension, 37 for diabetes, 17 for heart disease, 93 for dyslipidaemia, 25 for kidney disease, 23 for hypertension medications, 38 for diabetes medications, 96 for dyslipidaemia medications, 35 for systolic blood pressure, 35 for diastolic blood pressure, 9 for LDL-C, 2 for the eGFR, and 19 for HbA1c

^c^The TyG-BMI was calculated by the formula ln[Triglyceride (mg/dl) × Fasting blood glucose (mg/dl)/2] × BMI (kg/m^2^)

^d^Cumulative TyG-BMI was calculated by the formula (TyG-BMI_2012_ + TyG-BMI_2015_)/2 × time_(2015−2012)_

When compared with Class 1, participants in the other classes were more likely to be older, female, and married; to reside in a rural setting; to have a middle or high school education, fewer current smokers and drinkers, and higher systolic and diastolic blood pressure; to have a higher prevalence of hypertension, diabetes, dyslipidaemia and heart disease; to have a history of medication use for hypertension, diabetes and dyslipidaemia; to have higher TC, LDL-C, and HbA1 levels; and to have lower HDL-C levels (Table 1).

### Odds ratios for incident stroke

During a median follow-up of 36.5 months between 2015 and 2018, 277 participants experienced incident stroke, and the incidence rate of stroke was 6.0%. Table 2 shows the associations between the change in TyG-BMI and incident stroke events. After adjusting for potential confounders (in Model 4), compared with Class 1, the adjusted ORs (95% CIs) for incident stroke were 1.01 (0.65–1.57) for Class 2, 1.62 (1.11–2.32) for Class 3, and 1.71 (1.01–2.89) for Class 4. The results were found when modelling the cumulative TyG-BMI as quantiles (Table 2), and the baseline characteristics of the participants according to the quantiles of cumulative TyG-BMI are shown in Additional file 1: Table S2. After adjusting for confounders (in Model 4), when compared with Quartile 1, the adjusted ORs (95% CIs) for incident stroke were 1.66 (1.11–2.50) for Quartile 2, 1.41 (0.91–2.17) for Quartile 3, and 1.36 (0.85–2.18) for Quartile 4. Notably, no statistically significant differences were observed between Quartiles 3, 4, and 1 (P for trend = 0.563). Moreover, ROC analyses (Additional file 1: Fig. S1) demonstrated that baseline TyG-BMI had greater accuracy in predicting stroke compared with TyG (AUC, 0.62 vs. 0.57; P < 0.001).

**Table 2:** Associations of different classes of the TyG-BMI with stroke incidence

	No. of events/total	Model 1^a^		Model 2^b^		Model 3^c^		Model 4^d^	
		OR (95% CI)	P value	OR (95% CI)	P value	OR (95% CI)	P value	OR (95% CI)	P value
Change in the TyG-BMI^e^									
Class 1	48/1273	Reference		Reference		Reference		Reference	
Class 2	112/1664	1.64 (1.11–2.42)	0.014	1.68 (1.13–2.51)	0.010	1.17 (0.77–1.77)	0.463	1.01 (0.65–1.57)	0.965
Class 3	64/1171	1.98 (1.39–2.81)	< 0.001	2.03 (1.43–2.89)	< 0.001	1.74 (1.22–2.49)	0.002	1.62 (1.11–2.32)	0.011
Class 4	53/475	3.66 (2.41–5.56)	< 0.001	3.91 (2.55–5.98)	< 0.001	2.07 (1.29–3.31)	0.002	1.71 (1.01–2.89)	0.044
Cumulative TyG-BMI^f^									
Quartile 1 [353, 534]	41/1146	Reference		Reference		Reference		Reference	
Quartile 2 (534, 608]	73/1146	1.92 (1.30–2.85)	0.001	1.99 (1.34–2.95)	0.001	1.79 (1.20–2.67)	0.005	1.66 (1.11–2.50)	0.014
Quartile 3 (608, 693]	71/1145	1.91 (1.28–2.84)	0.002	2.06 (1.37–3.09)	< 0.001	1.60 (1.06–2.43)	0.027	1.41 (0.91–2.17)	0.122
Quartile 4 (693, 1130]	92/1146	2.56 (1.74–3.77)	< 0.001	2.80 (1.88–4.16)	< 0.001	1.65 (1.09–2.56)	0.022	1.36 (0.85–2.18)	0.205
P for trend^g^			< 0.001		< 0.001		0.102		0.563

BMI: body mass index; CI: confidence interval; OR: odds ratio; TyG: triglyceride-glucose

^a^Adjusted for age and sex

^b^Adjusted for age, sex, marital status, residence, educational level, smoking status, and drinking status

^c^Adjusted for variables in Model 2 and history of hypertension, diabetes, heart disease, dyslipidaemia, kidney disease, medication use for hypertension, medication use for diabetes, medication use for dyslipidaemia, systolic blood pressure, diastolic blood pressure

^d^Adjusted for variables in Model 3 and total cholesterol, HDL-C, LDL-C, HbA1c, and the eGFR

^e^The TyG-BMI was calculated by the formula ln[Triglyceride (mg/dl) × Fasting blood glucose (mg/dl)/2] × BMI (kg/m^2^), and the change in TyG-BMI from 2012 to 2015 was analysed and classified into 4 classes using K-means clustering

^f^The cumulative TyG-BMI was calculated by the formula (TyG-BMI_2012_ + TyG-BMI_2015_)/2 × time_(2015−2012)_, and then it was split into quartiles

^g^Tests for linear trends were performed by modelling the median value of each quantile to test ordered relations across quantiles of the cumulative TyG-BMI

**Fig. 3:**
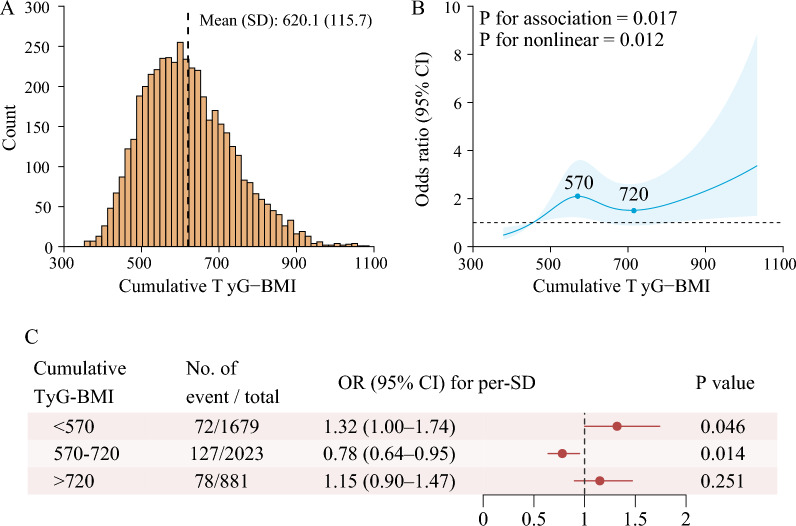
Nonlinear association between cumulative TyG-BMI and stroke. **A** Distribution for cumulative TyG-BMI from 2012 to 2015; **B** graphs show ORs for stroke. Data were fitted by a restricted cubic spline (RCS) logistics regression model, and the models were conducted with 4 knots at the 5th, 35th, 65th, and 95th percentiles of the cumulative TyG-BMI (reference is the 5th percentile). TyG-BMI ranged from 374.60 to 1032.59, due the RCS model handles outliers or extremes. Solid lines indicate ORs, and shadow shapes indicate 95% CIs. **C** Forest plot for the association between cumulative TyG-BMI and stroke. OR was evaluated by a 1‑SD increase in TyG-BMI. All models were adjusted for age, sex, marital status, residence, educational level, smoking status, drinking status, history of hypertension, diabetes, heart disease, dyslipidaemia, kidney disease, medication use for hypertension, medication use for diabetes, medication use for dyslipidaemia, systolic blood pressure, diastolic blood pressure, total cholesterol, HDL-C, LDL-C, HbA1c, and the eGFR. BMI: body mass index; CI: confidence interval; SD: standard deviation; OR: odds ratio; TyG: triglyceride-glucose

In the RCS model, the association between cumulative TyG-BMI and risk of incident stroke was nonlinear (for association, P = 0.017; for nonlinearity, P = 0.012) (Fig. 3). The risk of stroke increased with a cumulative TyG-BMI < 570 (OR per SD increased: 1.32, 95% CI 1.00–1.74). The risk decreased when the cumulative TyG-BMI was between 570 and 720 (OR per SD decreased: 0.78, 95% CI 0.64–0.95). The risk increased with a cumulative TyG-BMI > 720 (OR per SD increased: 1.15, 95% CI 0.90–1.47); however, the difference was not statistically significant (P = 0.251).

### Subgroup analyses

Tables 3 and 4 show the association of the change in TyG-BMI and cumulative TyG-BMI with incident stroke events stratified by potential risk factors. After adjusting for potential confounders, no interaction was found between changes in TyG-BMI classes and subgroup variables. Marital status and educational level moderated the association of cumulative TyG-BMI with stroke.

**Table 3:** Associations of different classes of change in TyG-BMI with stroke incidence stratified by different factors

Subgroup	Change in the TyG-BMI, OR (95% CI)				P for interaction
	Class 1 (n = 1273)	Class 2 (n = 1664)	Class 3 (n = 1171)	Class 4 (n = 475)	
Age (years)					0.192
< 60	Reference	1.22 (0.59–2.54)	2.07 (1.09–3.93)	2.65 (1.18–5.92)	
≥ 60	Reference	0.92 (0.52–1.64)	1.45 (0.91–2.28)	1.16 (0.54–2.51)	
Sex					0.919
Male	Reference	1.16 (0.60–2.25)	1.54 (0.92–2.59)	1.83 (0.78–4.30)	
Female	Reference	0.88 (0.47–1.62)	1.60 (0.93–2.73)	1.63 (0.82–3.25)	
Marital status					0.122
Married	Reference	1.25 (0.74–2.09)	1.95 (1.25–3.04)	1.90 (1.04–3.45)	
Other	Reference	0.49 (0.18–1.33)	1.08 (0.53–2.17)	1.45 (0.41–5.19)	
Residence					0.319
Urban	Reference	0.93 (0.34–2.59)	2.49 (0.99–6.26)	1.79 (0.58–5.50)	
Rural	Reference	1.07 (0.65–1.78)	1.42 (0.94–2.14)	1.60 (0.86–2.98)	
Educational level					0.153
No formal education	Reference	0.80 (0.37–1.73)	1.15 (0.63–2.10)	1.35 (0.53–3.43)	
Primary school	Reference	1.03 (0.53–2.02)	1.84 (1.06–3.20)	1.57 (0.68–3.59)	
Middle or high school	Reference	2.50 (0.75–8.31)	3.64 (1.19–11.16)	4.46 (1.19–16.69)	
College or above	Reference	NA	NA	NA	
Smoking status					0.999
Never	Reference	0.91 (0.51–1.65)	1.59 (0.95–2.66)	1.62 (0.82–3.19)	
Former	Reference	0.95 (0.24–3.75)	1.29 (0.42–4.00)	2.16 (0.43–10.78)	
Current	Reference	1.29 (0.55–3.00)	1.60 (0.85–2.99)	1.88 (0.64–5.50)	
Drinking status					0.860
Never	Reference	0.99 (0.56–1.77)	1.48 (0.90–2.44)	1.42 (0.72–2.83)	
Former	Reference	0.52 (0.12–2.21)	1.23 (0.39–3.88)	0.76 (0.11–5.52)	
Current	Reference	1.12 (0.49–2.57)	1.87 (0.98–3.58)	2.46 (0.92–6.56)	
Hypertension					0.540
No	Reference	1.03 (0.58–1.83)	1.49 (0.97–2.29)	2.06 (1.00–4.27)	
Yes	Reference	1.20 (0.53–2.74)	2.24 (1.04–4.83)	1.78 (0.72–4.39)	
Diabetes					0.556
No	Reference	1.01 (0.64–1.59)	1.65 (1.14–2.39)	1.73 (0.99–3.02)	
Yes	Reference	0.75 (0.06–9.83)	0.72 (0.06–9.34)	1.09 (0.07–15.94)	
Heart disease					0.752
No	Reference	0.96 (0.59–1.56)	1.50 (1.02–2.23)	1.74 (0.98–3.10)	
Yes	Reference	1.12 (0.34–3.75)	2.23 (0.76–6.60)	1.67 (0.43–6.44)	
Dyslipidaemia					0.529
No	Reference	0.91 (0.57–1.46)	1.54 (1.05–2.25)	1.79 (1.00–3.22)	
Yes	Reference	2.68 (0.31–23.49)	3.99 (0.47–33.50)	3.73 (0.40–34.69)	
Kidney disease					0.865
No	Reference	0.94 (0.60–1.49)	1.55 (1.06–2.26)	1.68 (0.98–2.88)	
Yes	Reference	2.20 (0.31–15.64)	3.22 (0.49–21.26)	3.22 (0.24–43.38)	

BMI: body mass index; CI: confidence interval; OR: odds ratio; TyG: triglyceride-glucose; NA: not applicable

All models were adjusted for age, sex, marital status, residence, educational level, smoking status, drinking status, history of hypertension, diabetes, heart disease, dyslipidaemia, kidney disease, medication use for hypertension, medication use for diabetes, medication use for dyslipidaemia, systolic blood pressure, diastolic blood pressure, total cholesterol, HDL-C, LDL-C, HbA1c, and the eGFR

**Table 4:** Associations of the cumulative TyG-BMI with stroke stratified by different factors

Subgroup	Cumulative TyG-BMI, OR (95% CI)				P for trend^a^	P for interaction
	Quartile 1 [353, 534]	Quartile 2 (534, 608]	Quartile 3 (608, 693]	Quartile 4 (693, 1130]		
Age (years)						0.151
< 60	Reference	2.59 (1.20–5.56)	2.14 (0.97–4.70)	2.43 (1.07–5.49)	0.188	
≥ 60	Reference	1.41 (0.86–2.32)	1.20 (0.70–2.08)	0.96 (0.51–1.81)	0.735	
Sex						0.354
Male	Reference	1.74 (0.97–3.12)	2.12 (1.14–3.94)	1.41 (0.66–3.01)	0.339	
Female	Reference	1.46 (0.82–2.60)	0.93 (0.51–1.72)	1.14 (0.61–2.14)	0.860	
Marital status						0.008
Married	Reference	2.04 (1.22–3.39)	2.04 (1.21–3.44)	1.76 (1.00–3.10)	0.256	
Other	Reference	1.25 (0.60–2.58)	0.42 (0.15–1.17)	0.65 (0.23–1.78)	0.219	
Residence						0.720
Urban	Reference	2.11 (0.75–5.95)	1.52 (0.54–4.32)	1.06 (0.35–3.22)	0.285	
Rural	Reference	1.53 (0.97–2.40)	1.39 (0.85–2.27)	1.43 (0.83–2.45)	0.337	
Educational level						0.034
No formal education	Reference	1.19 (0.61–2.33)	0.93 (0.44–1.97)	1.33 (0.60–2.96)	0.564	
Primary school	Reference	1.64 (0.89–3.04)	1.69 (0.89–3.22)	1.23 (0.59–2.54)	0.863	
Middle or high school	Reference	6.49 (1.44–29.29)	5.13 (1.12–23.55)	4.56 (0.94–22.07)	0.464	
College or above	Reference	NA	NA	NA	NA	
Smoking status						0.483
Never	Reference	1.54 (0.88–2.69)	1.00 (0.55–1.81)	1.20 (0.65–2.21)	0.924	
Former	Reference	0.89 (0.23–3.43)	1.85 (0.52–6.63)	1.57 (0.36–6.84)	0.444	
Current	Reference	1.91 (0.96–3.83)	2.33 (1.09–4.96)	1.23 (0.46–3.26)	0.476	
Drinking status						0.906
Never	Reference	1.55 (0.89–2.70)	1.30 (0.72–2.32)	1.26 (0.68–2.35)	0.865	
Former	Reference	1.61 (0.47–5.48)	0.65 (0.16–2.68)	0.75 (0.16–3.62)	0.503	
Current	Reference	1.83 (0.90–3.74)	1.90 (0.88–4.12)	1.72 (0.71–4.16)	0.307	
Hypertension						0.241
No	Reference	1.40 (0.87–2.25)	1.39 (0.82–2.35)	1.51 (0.83–2.74)	0.209	
Yes	Reference	3.24 (1.27–8.25)	2.16 (0.85–5.53)	1.82 (0.69–4.80)	0.644	
Diabetes						0.333
No	Reference	1.69 (1.12–2.56)	1.49 (0.96–2.31)	1.38 (0.84–2.26)	0.479	
Yes	Reference	1.00 (0.07–14.33)	0.24 (0.01–4.70)	0.78 (0.05–11.40)	0.927	
Heart disease						0.709
No	Reference	1.53 (0.99–2.36)	1.36 (0.85–2.16)	1.32 (0.79–2.20)	0.535	
Yes	Reference	2.52 (0.74–8.58)	1.58 (0.44–5.72)	1.62 (0.44–6.00)	0.991	
Dyslipidaemia						0.591
No	Reference	1.59 (1.05–2.43)	1.38 (0.88–2.18)	1.28 (0.77–2.12)	0.626	
Yes	Reference	4.38 (0.49–38.93)	2.78 (0.32–24.24)	2.92 (0.33–25.81)	0.983	
Kidney disease						0.066
No	Reference	1.48 (0.97–2.24)	1.30 (0.84–2.02)	1.26 (0.78–2.04)	0.659	0.151
Yes	Reference	NA	NA	NA	NA	

BMI: body mass index; CI: confidence interval; OR: odds ratio; TyG: triglyceride-glucose; NA: not applicable

All models were adjusted for age, sex, marital status, residence, educational level, smoking status, drinking status, history of hypertension, diabetes, heart disease, dyslipidaemia, kidney disease, medication use for hypertension, medication use for diabetes, medication use for dyslipidaemia, systolic blood pressure, diastolic blood pressure, total cholesterol, HDL-C, LDL-C, HbA1c, and the eGFR

^a^Tests for linear trends were performed by modelling the median value of each quantile to test ordered relations across quantiles of cumulative TyG-BMI

### WQS analyses

**Fig. 4:**
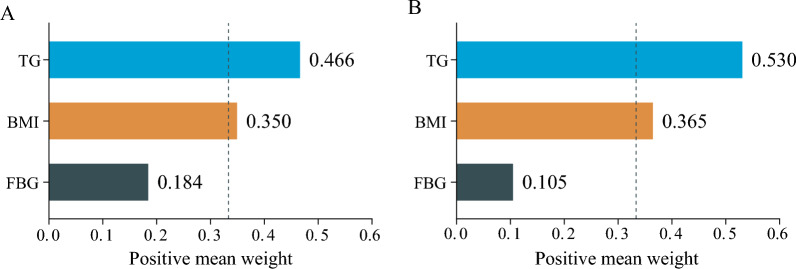
Estimated weights assigned to TyG-BMI with the WQS model. Weights in a positive direction obtained when the effect parameter of the WQS model was constrained to the positive direction with 1 000 repeated holdout validations for TyG-BMI in 2012 (**A**) and 2015 (**B**). WQS models were adjusted for age, sex, marital status, residence, educational level, smoking status, drinking status, history of hypertension, diabetes, heart disease, dyslipidaemia, kidney disease, medication use for hypertension, medication use for diabetes, medication use for dyslipidaemia, systolic blood pressure, diastolic blood pressure, total cholesterol, HDL-C, LDL-C, HbA1c, and the eGFR. BMI: body mass index; FBG: fasting blood glucose; TyG: triglyceride-glucose; WQS: weighted quantile sum

The WQS regression model was utilized to offer a thorough elucidation of the TyG-BMI. The weights assigned to the constituent elements of TyG-BMI, encompassing the cumulative influence of stroke while controlling for potential confounding variables, are presented in Fig. 4**.** Importantly, TG emerged as the primary contributor in both 2012 and 2015, with weights of 0.466 and 0.530, respectively.

### Sensitivity analyses

Sensitivity analyses consistently yielded congruent results when performing complete data analyses (Additional file 1: Table S3), excluding participants who had heart disease (Additional file 1: Table S4), employing competing risk regression to account for competing risks due to mortality (Additional file 1: Table S5), or employing Cox regression to account for censored data (Additional file 1: Table S6). Furthermore, the nonlinear nature of the association between cumulative TyG-BMI and the risk of incident stroke persisted when subjecting the data to complete data analyses (Additional file 1: Fig. S2) or treating cumulative TyG-BMI as a continuous variable (Additional file 1: Table S7).

## Discussion

Our study revealed that substantial changes in the TyG-BMI are independently associated with the risk of stroke in individuals aged 45 years and above from the CHARLS national data. Notably, the association between the cumulative TyG-BMI and stroke occurrence exhibited a nonlinear pattern; specifically, the risk of stroke increased with a cumulative TyG-BMI < 570. Considering that the TyG-BMI incorporates FBG, TG, and BMI, our investigation identified TG as the primary contributor to the observed association. Meanwhile, our findings have important clinical implications, suggesting that monitoring long-term changes in the TyG-BMI may assist with the early identification of individuals at high risk of stroke. Furthermore, to mitigate the risk of stroke, prioritizing the management of TG levels may be worth considering.

A previous investigation provided evidence demonstrating the rapid induction of IR in humans through elevated levels of free fatty acids in plasma [31]. In addition, when exposed to high-glucose conditions, glucose molecules form conjugates with proteins resembling the insulin receptor on the cytoplasmic membrane, resulting in the prompt formation of advanced glycation end products [32]. Consequently, the binding of serum insulin to the deposited advanced glycation end products on the insulin receptor becomes imperfect, thereby impeding the mediation of insulin's glucose transport stimulation and triggering IR [33]. Recent studies have suggested that the product of plasma TyG holds promise as an effective measure for detecting IR [6, 34]. Moreover, BMI, a straightforwards anthropometric parameter commonly employed as an indicator of obesity and IR, is noteworthy. In individuals with obesity, adipose tissue lipolysis intensifies, leading to the release of substantial amounts of free fatty acids, which represents a crucial factor influencing insulin sensitivity modulation [35]. Consequently, it is reasonable to hypothesize that the TyG-BMI, derived from the anthropometric BMI and TyG parameters, may also serve as a valuable marker for IR.

Indeed, the association between the TyG-BMI and HOMA-IR has been empirically established [13]. Consequently, the TyG-BMI has been advocated as a reliable metric for assessing IR and IR-related ailments in numerous studies. A cross-sectional investigation carried out in rural Beijing, China affirmed that the TyG-BMI exhibited superior efficacy in detecting IR [36]. Moreover, the 2015 Health, Well-Being, and Ageing Study proposed the utility of TyG-BMI as an assessment tool for prediabetes, albeit not as the optimal index [37]. Findings from a substantial cross-sectional survey involving 11,149 participants, the Korean National Health and Nutrition Examination Survey, demonstrated that TyG-BMI served as a viable alternative marker for evaluating IR when compared to other IR parameters [38]. Additionally, the associations between TyG-BMI and prehypertension, as well as hypertension, have been conclusively validated [39, 40]. Despite the identification of an association between TyG-BMI and ischaemic stroke in a recent cross-sectional study [19], certain limitations, such as the study's cross-sectional design and focus on stroke subtype, warrant caution in drawing definitive conclusions. Consequently, the precise association between TyG-BMI and ischaemic stroke remains equivocal.

Based on our current understanding, this study represents a novel approach in utilizing cluster analysis to categorize the changes in the TyG-BMI values. Each category within the analysis corresponded to distinct subpopulations, wherein individuals with a consistently low TyG-BMI exhibited the lowest risk, while those with a highest TyG-BMI and a slow declining trend displayed the highest risk. Previous studies predominantly relied on a single TyG-BMI value to predict the occurrence of stroke, often yielding different results on different occasions [19]. Furthermore, it is worth noting that our research sample consisted of a representative cross-section of healthy individuals from various regions of China. By focusing on dynamic processes, our investigation has contributed further evidence to elucidate the association between TyG-BMI and stroke. Specifically, our RCS model elucidated a nonlinear association between the cumulative TyG-BMI and stroke. However, the underlying mechanistic explanation for this observed association remains uncertain. Finally, we employed the WQS regression method to augment the interpretability of the TyG-BMI, wherein we observed that TG emerged as the primary contributor to the observed effects.

The precise mechanism by which IR contributes to the development of stroke remains poorly elucidated, with several potential pathways warranting consideration. First, IR is believed to induce endothelial dysfunction, foam cell formation, and the formation of vulnerable plaques, thereby playing a critical role in the pathogenesis of atherosclerosis [41–44]. Additionally, IR, characterized by a low-grade inflammatory state, facilitates the progression of atherosclerosis and stimulates the production of inflammatory markers [45, 46]. Second, IR exerts an impact on platelet adhesion, activation, and aggregation [47–50], culminating in stroke occurrence through arterial stenosis or occlusion. Third, IR has been associated with heightened sympathetic nervous system activity [34] and impaired cardiac autonomic function [51], both of which contribute to the pathophysiology of acute cardiovascular and cerebrovascular diseases. Last, individuals with IR often exhibit a larger waist circumference and BMI, hypertension, diabetes, cardiovascular disease, and a history of dyslipidaemia. Moreover, they frequently present with elevated fasting blood glucose levels, triglycerides, and glycosylated haemoglobin, all of which represent established risk factors for stroke [51–54].

Our study contributes to the existing body of knowledge by providing evidence supporting the utilization of the dynamic change in the TyG-BMI as a clinically valuable marker for identifying individuals at a heightened risk of cardiovascular disease. The TyG-BMI was derived through the calculation of FBG, TG, and BMI. However, apart from measuring TG and FBG, body weight and height are also needed to calculate the TyG-BMI, which results in a more complicated formula. We still assert that the TyG-BMI holds promising prospects for accurately identifying patients at a heightened risk of stroke. First, these biochemical parameters can be conveniently obtained from a single sample at the same time, presenting a cost-effective and convenient alternative to the euglycaemic-hyperinsulinaemic clamp method. Furthermore, the widespread availability and routine performance of height, weight, FBG, and TG measurements in primary healthcare settings enhance the generalizability and practicality of the TyG-BMI in both clinical and epidemiological studies. Second, the TyG-BMI is designed to offer a more comprehensive assessment of insulin resistance, particularly in populations where obesity is prevalent. By incorporating BMI, which reflects body adiposity, the TyG-BMI provides a more holistic evaluation that accounts for the influence of obesity on IR [13]. Third, obesity is a well-established risk factor for IR and metabolic disorders [14, 55]. In clinical practice, addressing weight-related factors is often a key aspect of managing IR. The TyG-BMI aligns with this clinical relevance, potentially guiding interventions and treatment strategies for individuals with obesity-related IR. Last, the ROC analysis demonstrated that baseline TyG-BMI had greater accuracy in predicting stroke compared with TyG.

However, it is essential to address several limitations associated with this study. First, while the TyG-BMI has demonstrated reliability and convenience as a surrogate marker for insulin resistance, establishing a direct association between IR and stroke necessitates a comparison with the gold standard diagnostic method. Unfortunately, this study did not undertake such a comparison, thereby limiting its ability to provide a direct explanation of the insulin resistance-stroke association. Second, the study only incorporated two blood tests, precluding a comprehensive assessment and refinement of the TyG-BMI. Third, it should be noted that, similar to other studies, the diagnosis of stroke in this research relied on self-reporting, which introduces a logistical constraint. Due to the absence of medical records in the CHARLS dataset, validation and confirmation of self-reported incident stroke were not feasible. However, it is worth mentioning that other large-scale studies, such as the English Longitudinal Study of Ageing, have reported a satisfactory level of agreement between self-reported incident stroke and medical records [56]. Fourth, this study adopts an observational design, and it is essential to acknowledge the presence of selection bias arising from the loss to follow-up. This bias is influenced by factors such as nonresponse due to severe cardiovascular disease or competing events resulting from mortality, which may potentially lead to an underestimation of the association between the TyG-BMI and stroke. Nevertheless, it is noteworthy that our findings remained consistent even after we conducted exclusions involving participants with pre-existing heart disease. Furthermore, we employed Cox regression or competing risk regression analyses, which also yielded results consistent with our primary findings. Last, only participants from China were involved in this study; thus, the findings may not be fully generalizable to other countries.

## Conclusions

In this study, we revealed that substantial changes in the TyG-BMI are independently associated with the risk of stroke in individuals aged 45 years and above from the CHARLS national data. Consequently, monitoring long-term changes in the TyG-BMI should prioritize stroke prevention strategies. Furthermore, our findings elucidated the underlying mechanisms of the TyG-BMI by highlighting TG as the primary contributor to the observed effects.

### Supplementary Information

**Additional file 1: Figure S1**. Receiver operating characteristic curves (ROCs) for baseline TyG-BMI predicting stroke. **Figure S2.** Nonlinear association between cumulative TyG-BMI and stroke in subpopulations of 4373 participants with complete data. **Table S1.** Baseline characteristics between participants included and not included. **Table S2.** Baseline characteristics of 4583 participants according to the quartile of cumulative TyG-BMI. **Table S3.** Associations of different classes of TyG-BMI with stroke incidence in subpopulations of 4373 participants with complete data. **Table S4.** Associations of different classes of TyG-BMI with stroke incidence in subpopulations of 4045 participants without heart disease. **Table S5.** Associations of different classes of TyG-BMI with stroke incidence using competing risk regression. **Table S6.** Associations of different classes of TyG-BMI with stroke incidence using the Cox proportional hazards regression. **Table S7.** Associations of cumulative TyG-BMI with stroke incidence when treating cumulative TyG-BMI as a continuous variable.

## Data Availability

Online repositories contain the datasets used in this investigation. The names of the repositories and accession numbers can be found at http://charls.pku.edu.cn/en.
