# Supplementary material for: Changes in the triglyceride glucose-body mass index estimate the risk of stroke in middle-aged and older Chinese adults: a nationwide prospective cohort study
Source: Cardiovasc Diabetol. 2023 Sep 16;22:254. doi: 10.1186/s12933-023-01983-5 (PMC10505325; doi:10.1186/s12933-023-01983-5)
Supplement: Supplementary file 1 — Additional file 1: Figure S1. Receiver operating characteristic curves (ROCs) for baseline TyG-BMI predicting stroke. Figure S2. Nonlinear association between cumulative TyG-BMI and stroke in subpopulations of 4373 participants with complete data. Table S1. Baseline characteristics between participants included and not included. Table S2. Baseline characteristics of 4583 participants according to the quartile of cumulative TyG-BMI. Table S3. Associations of different classes of TyG-BMI with stroke incidence in subpopulations of 4373 participants with complete data. Table S4. Associations of different classes of TyG-BMI with stroke incidence in subpopulations of 4045 participants without heart disease. Table S5. Associations of different classes of TyG-BMI with stroke incidence using competing risk regression. Table S6. Associations of different classes of TyG-BMI with stroke incidence using the Cox proportional hazards regression. Table S7. Associations of cumulative TyG-BMI with stroke incidence when treating cumulative TyG-BMI as a continuous variable. [file 12933_2023_1983_MOESM1_ESM.docx]

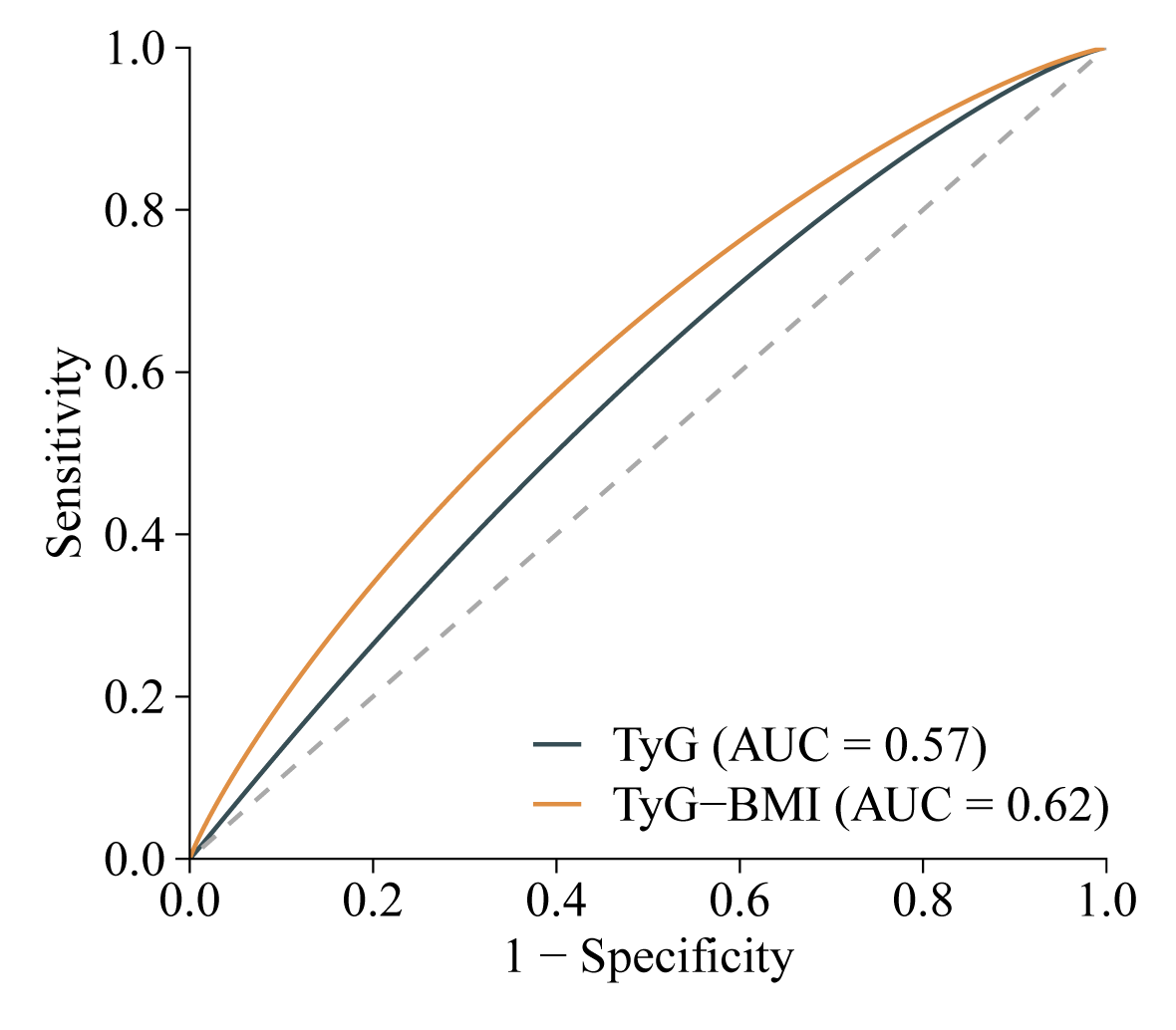


**Figure S1: Receiver operating characteristic curves (ROCs) for baseline TyG-BMI predicting stroke.**

Graphs show ROCs for baseline TyG and TyG-BMI predicting stroke, and the curves were generated with a smooth kernel density. Abbreviations:AUC, area under curve; BMI, body mass index; CI, confidence interval; TyG, triglyceride-glucose.

**
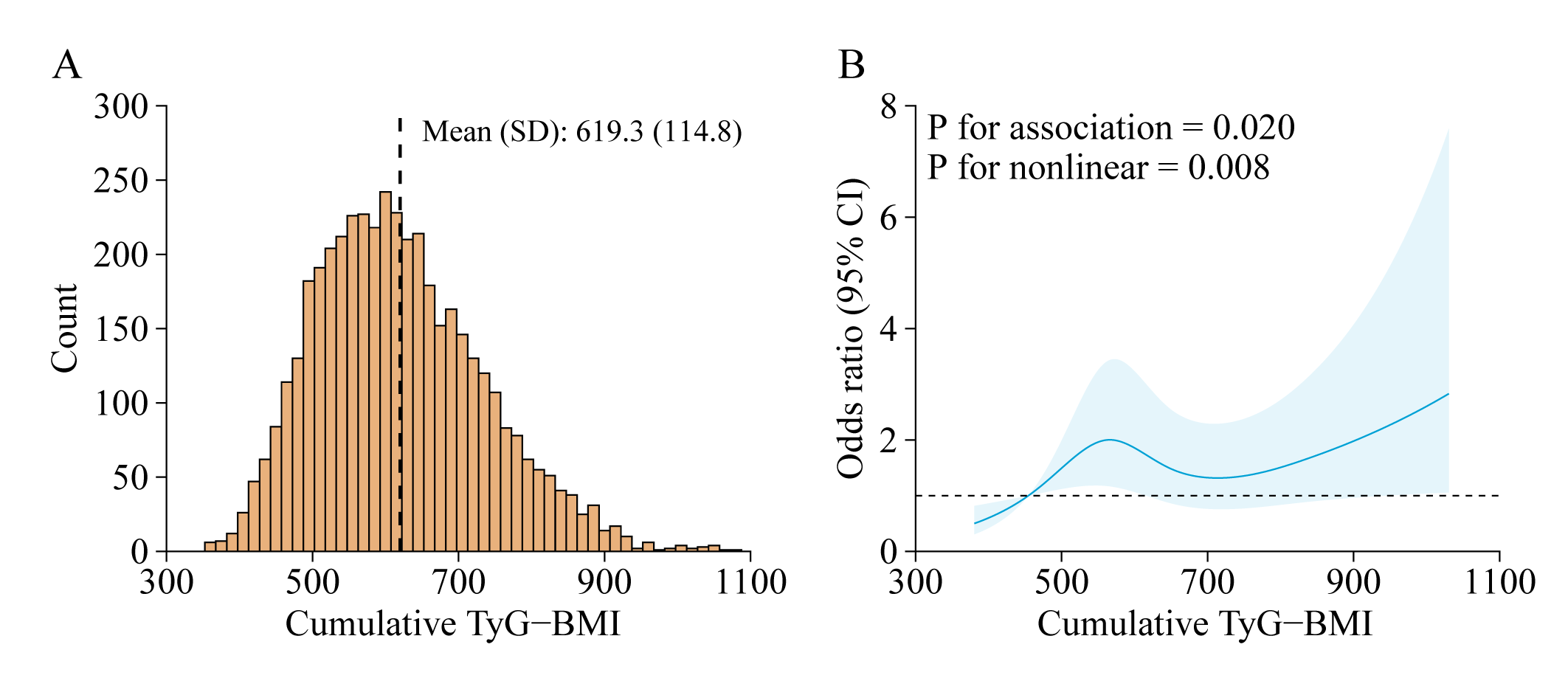
**

**Figure S2: Nonlinear association between** **cumulative TyG-BMI and stroke in subpopulations of 4 373 participants with complete data.**

(**A**) Distribution for cumulative TyG-BMI from 2012 to 2015; (**B**) Graphs show ORs for stroke. Data were fitted by a restricted cubic spline (RCS) logistics regression model, and the models were conducted with 4 knots at the 5^th^, 35^th^, 65^th^, and 95^th^ percentiles of the cumulative TyG-BMI (reference is the 5^th^ percentile). Solid lines indicate ORs, and shadow shapes indicate 95% CIs. Model was adjusted for age, sex, marital status, residence, educational level, smoking status, drinking status, history of hypertension, diabetes, heart disease, dyslipidaemia, kidney disease, medication use for hypertension, medication use for diabetes, medication use for dyslipidaemia, systole blood pressure, diastolic blood pressure, total cholesterol, HDL-C, LDL-C, HbA1c, and the eGFR. Abbreviations: BMI, body mass index; CI, confidence interval; OR, odds ratio; TyG, triglyceride-glucose.

**Table S1: Baseline characteristics between participants included and not included**

| Characteristic | Exclude (n=13125) | Include (n=4583) | P value ^a^ |
| --- | --- | --- | --- |
| Age, years |  |  | 0.123 |
| Mean±SD | 58.43±10.67 | 58.68±8.62 |  |
| Missing | 175 | 0 |  |
| Sex |  |  | <0.001 |
| Male | 6422 (48.9%) | 2056 (44.9%) |  |
| Female | 6701 (51.1%) | 2527 (55.1%) |  |
| Missing | 2 (0.0%) | 0 (0.0%) |  |
| Marital status |  |  | <0.001 |
| Married | 10265 (78.2%) | 3905 (85.2%) |  |
| Other | 2848 (21.7%) | 678 (14.8%) |  |
| Missing | 12 (0.1%) | 0 (0.0%) |  |
| Residence |  |  | <0.001 |
| Urban | 5643 (43.0%) | 1528 (33.3%) |  |
| Rural | 7482 (57.0%) | 3055 (66.7%) |  |
| Educational level |  |  | <0.001 |
| No formal education | 3507 (26.7%) | 1344 (29.3%) |  |
| Primary school | 5010 (38.2%) | 1910 (41.7%) |  |
| Middle or high school | 3896 (29.7%) | 1207 (26.3%) |  |
| College or above | 696 (5.3%) | 122 (2.7%) |  |
| Missing | 16 (0.1%) | 0 (0.0%) |  |
| Smoking status |  |  | 0.613 |
| Never | 7761 (59.1%) | 2858 (62.4%) |  |
| Former | 1043 (7.9%) | 374 (8.2%) |  |
| Current | 3530 (26.9%) | 1341 (29.3%) |  |
| Missing | 791 (6.0%) | 10 (0.2%) |  |
| Drinking status |  |  | 0.822 |
| Never | 7633 (58.2%) | 2700 (58.9%) |  |
| Former | 1076 (8.2%) | 367 (8.0%) |  |
| Current | 4254 (32.4%) | 1513 (33.0%) |  |
| Missing | 162 (1.2%) | 3 (0.1%) |  |
| Hypertension |  |  | 0.205 |
| No | 9591 (73.1%) | 3344 (73.0%) |  |
| Yes | 3319 (25.3%) | 1216 (26.5%) |  |
| Missing | 215 (1.6%) | 23 (0.5%) |  |
| Diabetes |  |  | 0.792 |
| No | 12081 (92.0%) | 4265 (93.1%) |  |
| Yes | 781 (6.0%) | 281 (6.1%) |  |
| Missing | 263 (2.0%) | 37 (0.8%) |  |
| Heart disease |  |  | 0.297 |
| No | 11308 (86.2%) | 4029 (87.9%) |  |
| Yes | 1593 (12.1%) | 537 (11.7%) |  |
| Missing | 224 (1.7%) | 17 (0.4%) |  |
| Dyslipidaemia |  |  | 0.005 |
| No | 11518 (87.8%) | 3999 (87.3%) |  |
| Yes | 1205 (9.2%) | 491 (10.7%) |  |
| Missing | 402 (3.1%) | 93 (2.0%) |  |
| History of medication use for hypertension |  |  | 0.773 |
| No | 10371 (79.0%) | 3675 (80.2%) |  |
| Yes | 2529 (19.3%) | 885 (19.3%) |  |
| Missing | 225 (1.7%) | 23 (0.5%) |  |
| History of medication use for diabetes |  |  | 0.193 |
| No | 12318 (93.9%) | 4374 (95.4%) |  |
| Yes | 541 (4.1%) | 171 (3.7%) |  |
| Missing | 266 (2.0%) | 38 (0.8%) |  |
| History of medication use for dyslipidaemia |  |  | 0.013 |
| No | 12106 (92.2%) | 4230 (92.3%) |  |
| Yes | 608 (4.6%) | 257 (5.6%) |  |
| Missing | 411 (3.1%) | 96 (2.1%) |  |
| Systolic blood pressure, mmHg |  |  | <0.001 |
| Mean±SD | 130.15±21.98 | 128.44±20.63 |  |
| Missing | 3933 | 35 |  |
| Diastolic blood pressure, mmHg |  |  | 0.001 |
| Mean±SD | 75.72±12.38 | 75.00±11.98 |  |
| Missing | 3934 | 35 |  |
| TC, mg/dl |  |  | <0.001 |
| Mean±SD | 191.81±38.83 | 194.74±38.93 |  |
| Missing | 6053 | 0 |  |
| HDL-C, mg/dl |  |  | 0.021 |
| Mean±SD | 50.58±15.35 | 51.25±15.29 |  |
| Missing | 6045 | 0 |  |
| LDL-C, mg/dl |  |  | <0.001 |
| Mean±SD | 114.95±34.83 | 117.61±34.97 |  |
| Missing | 6057 | 9 |  |
| HbA1c |  |  | 0.007 |
| Mean±SD | 5.25±0.83 | 5.29±0.81 |  |
| Missing | 5983 | 19 |  |

Abbreviations: HbA1c, glycated hemoglobin; HDL-C, high-density lipoprotein cholesterol; LDL-C, low-density lipoprotein cholesterol; SD, standard deviation; TC, total cholesterol.

^a^ P value was based on χ^2^ or analysis of variance test where appropriate.

**Table S2: Baseline characteristics of 4583 participants according to the quartile of cumulative TyG-BMI**

| Characteristic | Cumulative TyG-BMI | | | | P value ^a^ |
| --- | --- | --- | --- | --- | --- |
|  | Quartile 1  [353,534] (n=1146) | Quartile 2  (534,608] (n=1146) | Quartile 3  (608,693] (n=1145) | Quartile 4  (693,1130] (n=1146) |  |
| Age, mean±SD, years | 61.11±9.00 | 59.06±8.66 | 57.72±8.14 | 56.82±8.04 | <0.001 |
| Sex |  |  |  |  | <0.001 |
| Male | 677 (59.1%) | 532 (46.4%) | 456 (39.8%) | 391 (34.1%) |  |
| Female | 469 (40.9%) | 614 (53.6%) | 689 (60.2%) | 755 (65.9%) |  |
| Marital status |  |  |  |  | <0.001 |
| Married | 943 (82.3%) | 953 (83.2%) | 999 (87.2%) | 1010 (88.1%) |  |
| Other | 203 (17.7%) | 193 (16.8%) | 146 (12.8%) | 136 (11.9%) |  |
| Residence |  |  |  |  | <0.001 |
| Urban | 259 (22.6%) | 344 (30.0%) | 439 (38.3%) | 486 (42.4%) |  |
| Rural | 887 (77.4%) | 802 (70.0%) | 706 (61.7%) | 660 (57.6%) |  |
| Educational level |  |  |  |  | <0.001 |
| No formal education | 354 (30.9%) | 346 (30.2%) | 336 (29.3%) | 308 (26.9%) |  |
| Primary school | 525 (45.8%) | 476 (41.5%) | 445 (38.9%) | 464 (40.5%) |  |
| Middle or high school | 248 (21.6%) | 293 (25.6%) | 328 (28.6%) | 338 (29.5%) |  |
| College or above | 19 (1.7%) | 31 (2.7%) | 36 (3.1%) | 36 (3.1%) |  |
| Smoking status ^b^ |  |  |  |  | <0.001 |
| Never | 552 (48.2%) | 712 (62.1%) | 764 (66.7%) | 830 (72.4%) |  |
| Former | 84 (7.3%) | 86 (7.5%) | 106 (9.3%) | 98 (8.6%) |  |
| Current | 507 (44.2%) | 345 (30.1%) | 272 (23.8%) | 217 (18.9%) |  |
| Drinking status ^b^ |  |  |  |  | <0.001 |
| Never | 576 (50.3%) | 677 (59.1%) | 689 (60.2%) | 758 (66.1%) |  |
| Former | 107 (9.3%) | 80 (7.0%) | 90 (7.9%) | 90 (7.9%) |  |
| Current | 462 (40.3%) | 388 (33.9%) | 365 (31.9%) | 298 (26.0%) |  |
| History of comorbidities |  |  |  |  |  |
| Hypertension ^b^ | 151 (13.2%) | 224 (19.5%) | 338 (29.5%) | 503 (43.9%) | <0.001 |
| Diabetes ^b^ | 24 (2.1%) | 47 (4.1%) | 61 (5.3%) | 149 (13.0%) | <0.001 |
| Heart disease ^b^ | 103 (9.0%) | 107 (9.3%) | 130 (11.4%) | 197 (17.2%) | <0.001 |
| Dyslipidaemia ^b^ | 38 (3.3%) | 73 (6.4%) | 125 (10.9%) | 255 (22.3%) | <0.001 |
| Kidney disease ^b^ | 60 (5.2%) | 61 (5.3%) | 61 (5.3%) | 74 (6.5%) | 0.536 |
| History of medication use |  |  |  |  |  |
| Hypertension medications ^b^ | 95 (8.3%) | 150 (13.1%) | 237 (20.7%) | 403 (35.2%) | <0.001 |
| Diabetes medications ^b^ | 17 (1.5%) | 29 (2.5%) | 24 (2.1%) | 101 (8.8%) | <0.001 |
| Dyslipidaemia medications ^b^ | 16 (1.4%) | 35 (3.1%) | 64 (5.6%) | 142 (12.4%) | <0.001 |
| Blood pressure, mean±SD, mmHg |  |  |  |  |  |
| Sytolic ^b^ | 123.95±20.07 | 125.82±19.46 | 129.68±20.33 | 134.32±21.11 | <0.001 |
| Diastolic ^b^ | 71.42±11.21 | 73.07±11.64 | 76.11±11.33 | 79.43±12.18 | <0.001 |
| TC, mean±SD, mg/dl | 185.25±36.19 | 191.45±36.96 | 195.64±36.48 | 206.63±42.63 | <0.001 |
| HDL-C, mean±SD, mg/dl | 59.97±16.41 | 53.60±14.06 | 48.57±12.57 | 42.85±12.27 | <0.001 |
| LDL-C, mean±SD, mg/dl ^b^ | 110.16±31.20 | 118.18±32.88 | 120.58±34.09 | 121.54±40.02 | <0.001 |
| HbA1c, mean±SD | 5.14±0.58 | 5.19±0.61 | 5.24±0.75 | 5.59±1.11 | <0.001 |
| eGFR, mean±SD, ml/min/1.73m^2 b^ | 73.02±32.50 | 73.99±35.49 | 75.38±32.18 | 75.66±34.95 | 0.205 |
| TG_2012_, median (IQR) | 74.34 (58.41, 97.35) | 93.81 (70.80, 126.56) | 113.28 (84.07, 161.07) | 158.41 (115.05, 237.18) | <0.001 |
| TG_2015_, median (IQR) | 79.20 (64.60, 103.54) | 100.00 (78.76, 130.75) | 128.32 (97.35, 173.45) | 172.57 (128.32, 247.79) | <0.001 |
| FBG_2012_, mean±SD | 101.84±21.96 | 104.52±22.84 | 108.69±32.45 | 123.39±48.62 | <0.001 |
| FBG_2015_, mean±SD | 92.34±18.54 | 96.21±22.42 | 101.18±28.02 | 115.17±45.61 | <0.001 |
| BMI_2012_, mean±SD | 19.60±1.67 | 22.34±1.40 | 24.46±1.66 | 28.20±3.10 | <0.001 |
| BMI_2015_, mean±SD | 19.65±1.72 | 22.59±1.45 | 24.81±1.71 | 28.20±2.82 | <0.001 |
| TyG-BMI_2012_, mean±SD ^c^ | 161.59±14.52 | 189.58±11.87 | 213.58±14.02 | 259.14±30.35 | <0.001 |
| TyG-BMI_2015_, mean±SD ^c^ | 161.63±15.04 | 191.59±11.69 | 217.84±14.47 | 258.71±27.52 | <0.001 |

Abbreviations: BMI, body mass index; eGFR, estimated glomerular filtration ratio; FBG, fast blood glucose; HbA1c, glycated hemoglobin; HDL-C, high-density lipoprotein cholesterol; IQR, interquartile range; LDL-C, low-density lipoprotein cholesterol; SD, standard deviation; TC, total cholesterol; TG, triglyceride; TyG, triglyceride-glucose.

^a^ P value was based on χ^2^ or analysis of variance test where appropriate.

^b^ Missing data: 10 for smoking status, 3 for drinking status, 23 for hypertension, 37 for diabetes, 17 for heart disease, 93 for dyslipidaemia, 25 for kidney disease, 23 for hypertension medications, 38 for diabetes medications, 96 for dyslipidaemia medications, 35 for systolic blood pressure, 35 for diastolic blood pressure, 9 for LDL-C, 2 for eGFR, and 19 for HbA1c.

^c^ The TyG-BMI was calculated by the formula ln[Triglyceride (mg/dl) × Fasting blood glucose (mg/dl) / 2] × BMI (kg/m^2^).

**Table S3: Associations of different classes of TyG-BMI with stroke incidence in subpopulations of 4 373 participants with complete data**

|  | No. of event / total | Model 1 ^a^ | |  | Model 2 ^b^ | |  | Model 3 ^c^ | |  | Model 4 ^d^ | |
| --- | --- | --- | --- | --- | --- | --- | --- | --- | --- | --- | --- | --- |
|  |  | OR (95% CI) | P value |  | OR (95% CI) | P value |  | OR (95% CI) | P value |  | OR (95% CI) | P value |
| Change in the TyG-BMI ^e^ |  |  |  |  |  |  |  |  |  |  |  |  |
| Class 1 | 48 / 1217 | Reference |  |  | Reference |  |  | Reference |  |  | Reference |  |
| Class 2 | 110 / 1595 | 1.54 (1.03–2.28) | 0.033 |  | 1.58 (1.05–2.35) | 0.028 |  | 1.09 (0.72–1.67) | 0.683 |  | 1.07 (0.70–1.63) | 0.765 |
| Class 3 | 61 / 1118 | 1.93 (1.35–2.74) | <0.001 |  | 1.98 (1.39–2.82) | <0.001 |  | 1.70 (1.19–2.44) | 0.004 |  | 1.68 (1.17–2.41) | 0.005 |
| Class 4 | 48 / 443 | 3.31 (2.16–5.08) | <0.001 |  | 3.54 (2.29–5.47) | <0.001 |  | 1.90 (1.18–3.08) | 0.009 |  | 1.85 (1.13–3.04) | 0.015 |
| Cumulative TyG-BMI ^f^ |  |  |  |  |  |  |  |  |  |  |  |  |
| Quartile 1 [353, 534] | 41 / 1096 | Reference |  |  | Reference |  |  | Reference |  |  | Reference |  |
| Quartile 2 (534, 608] | 72 / 1095 | 1.91 (1.29–2.84) | 0.001 |  | 1.95 (1.31–2.91) | 0.001 |  | 1.76 (1.18–2.63) | 0.006 |  | 1.61 (1.07–2.42) | 0.023 |
| Quartile 3 (608, 693] | 69 / 1101 | 1.88 (1.26–2.82) | 0.002 |  | 1.97 (1.31–2.96) | 0.001 |  | 1.53 (1.01–2.33) | 0.045 |  | 1.30 (0.84–2.01) | 0.245 |
| Quartile 4 (693, 1130] | 85 / 1081 | 2.44 (1.65–3.62) | <0.001 |  | 2.56 (1.71–3.83) | <0.001 |  | 1.53 (0.99–2.37) | 0.056 |  | 1.17 (0.72–1.90) | 0.514 |
| P for trend ^g^ |  |  | <0.001 |  |  | <0.001 |  |  | 0.230 |  |  | 0.921 |

Abbreviations: BMI, body mass index; CI, confidence interval; OR, odds ratio; TyG, triglyceride-glucose.

^a^ Adjusted for age, sex.

^b^ Adjusted for age, sex, marital status, residence, educational level, smoking status, and drinking status.

^c^ Adjusted for variables in model 2 and history of hypertension, diabetes, heart disease, dyslipidaemia, kidney disease, medication use for hypertension, medication use for diabetes, medication use for dyslipidaemia, systolic blood pressure, diastolic blood pressure.

^d^ Adjusted for variables in model 3 and total cholesterol, HDL-C, LDL-C, HbA1c, and the eGFR.

^e^ The TyG-BMI was calculated by the formula ln[Triglyceride (mg/dl) × Fasting blood glucose (mg/dl) / 2] × BMI (kg/m2), and the change of TyG-BMI from 2012 to 2015 was analysed and classified into 4 classes using K-means clustering.

^f^ The cumulative TyG-BMI was calculated by the formula (TyG-BMI_2012_+ TyG-BMI_2015_) / 2 × time(2015−2012), and then it was split into quartiles.

^g^ Tests for linear trends were done by modelling the median value of each quantile to test ordered relations across quantiles of the cumulative TyG-BMI.

**Table S4: Associations of different classes of TyG-BMI with stroke incidence in subpopulations of 4 045 participants without heart disease**

|  | No. of event / total | Model 1 ^a^ | |  | Model 2 ^b^ | |  | Model 3 ^c^ | |  | Model 4 ^d^ | |
| --- | --- | --- | --- | --- | --- | --- | --- | --- | --- | --- | --- | --- |
|  |  | OR (95% CI) | P value |  | OR (95% CI) | P value |  | OR (95% CI) | P value |  | OR (95% CI) | P value |
| Change in the TyG-BMI ^e^ |  |  |  |  |  |  |  |  |  |  |  |  |
| Class 1 | 43 / 1160 | Reference |  |  | Reference |  |  | Reference |  |  | Reference |  |
| Class 2 | 92 / 1500 | 1.54 (1.01–2.37) | 0.046 |  | 1.59 (1.03–2.45) | 0.036 |  | 1.19 (0.75–1.86) | 0.461 |  | 1.00 (0.62–1.62) | 0.991 |
| Class 3 | 50 / 1010 | 1.86 (1.28–2.71) | 0.001 |  | 1.91 (1.31–2.78) | 0.001 |  | 1.68 (1.15–2.47) | 0.008 |  | 1.53 (1.04–2.27) | 0.032 |
| Class 4 | 37 / 375 | 3.42 (2.13–5.47) | <0.001 |  | 3.66 (2.26–5.90) | <0.001 |  | 2.26 (1.35–3.80) | 0.002 |  | 1.82 (1.02–3.24) | 0.043 |
| Cumulative TyG-BMI ^f^ |  |  |  |  |  |  |  |  |  |  |  |  |
| Quartile 1 [353, 534] | 36 / 1012 | Reference |  |  | Reference |  |  | Reference |  |  | Reference |  |
| Quartile 2 (534, 608] | 59 / 1011 | 1.80 (1.18–2.76) | 0.007 |  | 1.83 (1.19–2.82) | 0.006 |  | 1.70 (1.10–2.62) | 0.016 |  | 1.57 (1.01–2.43) | 0.045 |
| Quartile 3 (608, 693] | 57 / 1011 | 1.84 (1.19–2.85) | 0.006 |  | 1.93 (1.24–3.00) | 0.004 |  | 1.59 (1.01–2.48) | 0.044 |  | 1.38 (0.86–2.20) | 0.179 |
| Quartile 4 (693, 1130] | 70 / 1011 | 2.36 (1.54–3.62) | <0.001 |  | 2.48 (1.61–3.83) | <0.001 |  | 1.66 (1.04–2.64) | 0.033 |  | 1.31 (0.79–2.19) | 0.296 |
| P for trend ^g^ |  |  | <0.001 |  |  | <0.001 |  |  | 0.094 |  |  | 0.583 |

Abbreviations: BMI, body mass index; CI, confidence interval; OR, odds ratio; TyG, triglyceride-glucose.

^a^ Adjusted for age, sex.

^b^ Adjusted for age, sex, marital status, residence, educational level, smoking status, and drinking status.

^c^ Adjusted for variables in model 2 and history of hypertension, diabetes, dyslipidaemia, kidney disease, medication use for hypertension, medication use for diabetes, medication use for dyslipidaemia, systolic blood pressure, diastolic blood pressure.

^d^ Adjusted for variables in model 3 and total cholesterol, HDL-C, LDL-C, HbA1c, and the eGFR.

^e^ The TyG-BMI was calculated by the formula ln[Triglyceride (mg/dl) × Fasting blood glucose (mg/dl) / 2] × BMI (kg/m2), and the change of TyG-BMI from 2012 to 2015 was analysed and classified into 4 classes using K-means clustering.

^f^ The cumulative TyG-BMI was calculated by the formula (TyG-BMI_2012_+ TyG-BMI_2015_) / 2 × time(2015−2012), and then it was split into quartiles.

^g^ Tests for linear trends were done by modeling the median value of each quantile to test ordered relations across quantiles of the cumulative TyG-BMI.

**Table S5: Associations of different classes of TyG-BMI with stroke incidence using competing risk regression**

|  | No. of event / total | Model 1 ^a^ | |  | Model 2 ^b^ | |  | Model 3 ^c^ | |  | Model 4 ^d^ | |
| --- | --- | --- | --- | --- | --- | --- | --- | --- | --- | --- | --- | --- |
|  |  | HR (95% CI) | P value |  | HR (95% CI) | P value |  | HR (95% CI) | P value |  | HR (95% CI) | P value |
| Change in the TyG-BMI ^e^ |  |  |  |  |  |  |  |  |  |  |  |  |
| Class 1 | 48 / 1273 | Reference |  |  | Reference |  |  | Reference |  |  | Reference |  |
| Class 2 | 112 / 1664 | 1.66 (1.15–2.41) | 0.007 |  | 1.69 (1.16–2.46) | 0.007 |  | 1.27 (0.87–1.85) | 0.223 |  | 1.09 (0.73–1.63) | 0.660 |
| Class 3 | 64 / 1171 | 2.00 (1.44–2.80) | <0.001 |  | 2.04 (1.46–2.84) | <0.001 |  | 1.79 (1.28–2.50) | 0.001 |  | 1.66 (1.19–2.33) | 0.003 |
| Class 4 | 53 / 475 | 3.61 (2.45–5.31) | <0.001 |  | 3.76 (2.55–5.53) | <0.001 |  | 2.13 (1.39–3.26) | <0.001 |  | 1.79 (1.12–2.88) | 0.016 |
| Cumulative TyG-BMI ^f^ |  |  |  |  |  |  |  |  |  |  |  |  |
| Quartile 1 [353, 534] | 41 / 1146 | Reference |  |  | Reference |  |  | Reference |  |  | Reference |  |
| Quartile 2 (534, 608] | 73 / 1146 | 1.93 (1.33–2.82) | 0.001 |  | 1.96 (1.35–2.86) | <0.001 |  | 1.79 (1.23–2.61) | 0.002 |  | 1.67 (1.14–2.45) | 0.008 |
| Quartile 3 (608, 693] | 71 / 1145 | 2.02 (1.38–2.97) | <0.001 |  | 2.07 (1.41–3.05) | <0.001 |  | 1.67 (1.13–2.47) | 0.010 |  | 1.48 (0.99–2.23) | 0.059 |
| Quartile 4 (693, 1130] | 92 / 1146 | 2.62 (1.82–3.79) | <0.001 |  | 2.71 (1.87–3.92) | <0.001 |  | 1.72 (1.17–2.52) | 0.006 |  | 1.42 (0.93–2.17) | 0.101 |
| P for trend ^g^ |  |  | <0.001 |  |  | <0.001 |  |  | 0.031 |  |  | 0.362 |

Abbreviations: BMI, body mass index; CI, confidence interval; OR, odds ratio; TyG, triglyceride-glucose.

^a^ Adjusted for age, sex.

^b^ Adjusted for age, sex, marital status, residence, educational level, smoking status, and drinking status.

^c^ Adjusted for variables in model 2 and history of hypertension, diabetes, heart disease, dyslipidaemia, kidney disease, medication use for hypertension, medication use for diabetes, medication use for dyslipidaemia, systolic blood pressure, diastolic blood pressure.

^d^ Adjusted for variables in model 3 and total cholesterol, HDL-C, LDL-C, HbA1c, and the eGFR.

^e^ The TyG-BMI was calculated by the formula ln[Triglyceride (mg/dl) × Fasting blood glucose (mg/dl) / 2] × BMI (kg/m2), and the change of TyG-BMI from 2012 to 2015 was analysed and classified into 4 classes using K-means clustering.

^f^ The cumulative TyG-BMI was calculated by the formula (TyG-BMI_2012_+ TyG-BMI_2015_) / 2 × time(2015−2012), and then it was split into quartiles.

^g^ Tests for linear trends were done by modelling the median value of each quantile to test ordered relations across quantiles of the cumulative TyG-BMI.

**Table S6: Associations of different classes of TyG-BMI with stroke incidence using the Cox proportional hazards regression**

|  | No. of event / total | Model 1 ^a^ | |  | Model 2 ^b^ | |  | Model 3 ^c^ | |  | Model 4 ^d^ | |
| --- | --- | --- | --- | --- | --- | --- | --- | --- | --- | --- | --- | --- |
|  |  | HR (95% CI) | P value |  | HR (95% CI) | P value |  | HR (95% CI) | P value |  | HR (95% CI) | P value |
| Change in the TyG-BMI ^e^ |  |  |  |  |  |  |  |  |  |  |  |  |
| Class 1 | 48 / 1273 | Reference |  |  | Reference |  |  | Reference |  |  | Reference |  |
| Class 2 | 112 / 1664 | 1.67 (1.14–2.45) | 0.008 |  | 1.69 (1.15–2.49) | 0.007 |  | 1.28 (0.86–1.90) | 0.231 |  | 1.10 (0.72–1.68) | 0.654 |
| Class 3 | 64 / 1171 | 2.03 (1.44–2.85) | <0.001 |  | 2.06 (1.46–2.90) | <0.001 |  | 1.82 (1.29–2.58) | 0.001 |  | 1.69 (1.19–2.41) | 0.003 |
| Class 4 | 53 / 475 | 3.70 (2.48–5.52) | <0.001 |  | 3.85 (2.56–5.79) | <0.001 |  | 2.22 (1.41–3.48) | 0.001 |  | 1.86 (1.13–3.06) | 0.015 |
| Cumulative TyG-BMI ^f^ |  |  |  |  |  |  |  |  |  |  |  |  |
| Quartile 1 [353, 534] | 41 / 1146 | Reference |  |  | Reference |  |  | Reference |  |  | Reference |  |
| Quartile 2 (534, 608] | 73 / 1146 | 1.95 (1.33–2.87) | 0.001 |  | 1.98 (1.35–2.91) | 0.001 |  | 1.82 (1.23–2.68) | 0.003 |  | 1.70 (1.15–2.52) | 0.008 |
| Quartile 3 (608, 693] | 71 / 1145 | 2.04 (1.38–3.02) | <0.001 |  | 2.09 (1.41–3.10) | <0.001 |  | 1.69 (1.13–2.52) | 0.010 |  | 1.49 (0.98–2.27) | 0.059 |
| Quartile 4 (693, 1130] | 92 / 1146 | 2.66 (1.82–3.89) | <0.001 |  | 2.75 (1.87–4.03) | <0.001 |  | 1.76 (1.16–2.65) | 0.007 |  | 1.45 (0.92–2.28) | 0.106 |
| P for trend ^g^ |  |  | <0.001 |  |  | <0.001 |  |  | 0.042 |  |  | 0.363 |

Abbreviations: BMI, body mass index; CI, confidence interval; HR, hazard ratio; TyG, triglyceride-glucose.

^a^ Adjusted for age, sex.

^b^ Adjusted for age, sex, marital status, residence, educational level, smoking status, and drinking status.

^c^ Adjusted for variables in model 2 and history of hypertension, diabetes, heart disease, dyslipidaemia, kidney disease, medication use for hypertension, medication use for diabetes, medication use for dyslipidaemia, systolic blood pressure, diastolic blood pressure.

^d^ Adjusted for variables in model 3 and total cholesterol, HDL-C, LDL-C, HbA1c, and the eGFR.

^e^ The TyG-BMI was calculated by the formula ln[Triglyceride (mg/dl) × Fast blood glucose (mg/dl) / 2] × BMI (kg/m2), and the change of TyG-BMI from 2012 to 2015 was analysed and classified into 4 classes using K-means clustering.

^f^ The cumulative TyG-BMI was calculated by the formula (TyG-BMI_2012_+ TyG-BMI_2015_) / 2 × time(2015−2012), and then it was split into quartiles.

^g^ Tests for linear trends were done by modelling the median value of each quantile to test ordered relations across quantiles of the cumulative TyG-BMI.

**Table S7: Associations of cumulative TyG-BMI with stroke incidence when treating cumulative TyG-BMI as a continuous variable**

| Cumulative TyG-BMI | Model 1 ^a^ | |  | Model 2 ^b^ | |  | Model 3 ^c^ | |  | Model 4 ^d^ | |
| --- | --- | --- | --- | --- | --- | --- | --- | --- | --- | --- | --- |
|  | OR (95% CI) | P value |  | OR (95% CI) | P value |  | OR (95% CI) | P value |  | OR (95% CI) | P value |
| Per 1 SD | 1.40 (1.25–1.58) | <0.001 |  | 1.43 (1.27–1.61) | <0.001 |  | 1.17 (1.02–1.34) | 0.025 |  | 1.10 (0.94–1.29) | 0.219 |

Abbreviations: BMI, body mass index; CI, confidence interval; OR, odds ratio; TyG, triglyceride-glucose.

^a^ Adjusted for age, sex.

^b^ Adjusted for age, sex, marital status, residence, educational level, smoking status, and drinking status.

^c^ Adjusted for variables in model 2 and history of hypertension, diabetes, heart disease, dyslipidaemia, kidney disease, medication use for hypertension, medication use for diabetes, medication use for dyslipidaemia, systolic blood pressure, diastolic blood pressure.

^d^ Adjusted for variables in model 3 and total cholesterol, HDL-C, LDL-C, HbA1c, and the eGFR.
